# Is preoperative ultrasound tumor size a prognostic factor in endometrial carcinoma patients?

**DOI:** 10.3389/fonc.2022.993629

**Published:** 2022-09-23

**Authors:** Marco Ambrosio, Antonio Raffone, Andrea Alletto, Chiara Cini, Francesco Filipponi, Daniele Neola, Matilde Fabbri, Alessandro Arena, Diego Raimondo, Paolo Salucci, Manuela Guerrini, Antonio Travaglino, Roberto Paradisi, Antonio Mollo, Renato Seracchioli, Paolo Casadio

**Affiliations:** ^1^ Mother-Child Department, Ospedale Maggiore, Azienda Unità Sanitaria Locale di Bologna, Bologna, Italy; ^2^ Division of Gynaecology and Human Reproduction Physiopathology, IRCCS Azienda Ospedaliero-Universitaria di Bologna, Bologna, Italy; ^3^ Department of Medical and Surgical Sciences, University of Bologna, Bologna, Italy; ^4^ Gynecology and Obstetrics Unit, Department of Neuroscience, Reproductive Sciences and Densitry, School of Medicine, University of Naples Federico II, Naples, Italy; ^5^ Gynecopathology and Breast Pathology Unit, Department of Woman’s Health Science, Agostino Gemelli University Polyclinic, Rome, Italy; ^6^ Gynecology and Obstetrics Unit, Department of Medicine, Surgery and Dentistry “Schola Medica Salernitana”, University of Salerno, Baronissi, Italy

**Keywords:** risk assessment, cancer, tumor, prognosis, death, recurrence, relapse

## Abstract

**Objective:**

We aimed to assess the prognostic value of preoperative ultrasound tumor size in EC through a single center, observational, retrospective, cohort study.

**Methods:**

Medical records and electronic clinical databases were searched for all consecutive patients with EC, preoperative ultrasound scans available to *ad hoc* estimate tumor size, and a follow-up of at least 2-year, at our Institution from January 2010 to June 2018. Patients were divided into two groups based on different dimensional cut-offs for the maximum tumor diameter: 2, 3 and 4 cm. Differences in overall survival (OS), disease specific survival (DSS) and progression-free survival (PFS) were assessed among the groups by using the Kaplan–Meier estimator and the log-rank test.

**Results:**

108 patients were included in the study. OS, DSS and PFS did not significantly differ between the groups based on the different tumor diameter cut-offs. No significant differences were found among the groups sub-stratified by age, BMI, FIGO stage, FIGO grade, lymphovascular space invasion status, myometrial invasion, lymph nodal involvement, histotype, and adjuvant treatment.

**Conclusions:**

Preoperative ultrasound tumor size does not appear as a prognostic factor in EC women.

## Highlights

Preoperative ultrasound assessment of tumor size in women with endometrial cancer does not seem to be a prognostic factor for OS, DSS or PFS.

## Introduction

Endometrial carcinoma (EC) is the most common gynecologic malignancy in western countries (1). In the last two decades, it has shown an increase in number of deaths even higher than that in incidence, because of an inaccurate risk stratification (1, 2).

In 2020, in order to improve such an inaccurate risk assessment, the ESGO-ESTRO-ESP guidelines for the management of EC patients recommended to integrate The Cancer Genome ATLAS (TCGA) molecular signature and conventional histological factors (3). In particular, EC patients are assigned to a risk group and therefore to a type of adjuvant treatment based on the International Federation of Gynecology and Obstetrics (FIGO) stage, histotype, FIGO grade, lymphonodal status, myometrial invasion depth, lymphovascular space invasion (LVSI) and molecular signature (i.e., DNA polymerase epsilon mutations, p53 abnormal expression and mismatch repair deficient expression) (3).

In accordance with the principles of the precision medicine, an increasingly tailored approach is recommended to improve survival in cancer patients (4–6), highlighting the need for adding new prognostic factors and integrating them with the current ones (3). In EC patients, the tumor size appears as one of the histological prognostic factors remained to be further investigated. In fact, although it has shown prognostic significance in several malignancies (7, 8), its value is unclear in EC patients. On the one hand, some studies suggested a prognostic value as it could affect the risk of lymph node metastasis (9–12). In particular, Schink et al. observed that tumors larger than 2 cm were associated with an increased risk of lymph node involvement (13). On the other hand, some studies reported that tumor size was not an independent prognostic factor as the rate of lymph node involvement was similar regardless of the size of the lesion (14–16).

The aim of this study was to assess the prognostic value of preoperative ultrasound tumor size in EC patients.

## Materials and methods

### Study protocol and patient selection

The study was carried out according to an *a priori* defined protocol, and was designed as a single center, observational, retrospective, cohort study.

The Strengthening the Reporting of Observational Studies in Epidemiology (STROBE) guidelines and checklist were followed for study reporting (17).

Medical records and electronic clinical databases were searched for all consecutive patients with histological diagnosis of EC after definitive surgery at our Institution from January 2010 to June 2018. Inclusion criteria were: patients with EC diagnosis; availability of stored preoperative ultrasound scans performed by an expert sonographer (i.e. sonographers with at least 5 years of experience in onco-gynecological ultrasound) to *ad hoc* estimate tumor size; follow-up of at least 2-year. Patients who did not undergo definitive surgery were excluded. No selection was made based on EC histological prognostic factors, FIGO stage or adjuvant treatment.

Based on the data available in the Literature, although the most common cut off used for the tumor diameter in EC patients was 2.0 cm (12, 13, 15, 18), some authors used greater cut-offs (11, 14, 19). Therefore, we divided our population into two groups according to different dimensional cut-offs for the maximum tumor diameter: 2.0, 3.0 and 4.0 cm. Differences in survival outcomes were assessed among the groups.

### Main outcome measures

The primary outcome measure was the difference in overall survival (OS) between patients with tumor ≥ and < 2 cm.

Secondary outcome measures were the difference in OS, disease specific survival (DSS) and progression-free survival (PFS) among the groups according to the different tumor diameter cut-offs.

The time of origin for patient survival was set as the date of surgery. In particular, OS was defined as time from surgery until death of any cause, DSS as time from surgery until death due to EC, and PFS as time from surgery until there was evidence of recurrent or progressive disease (diagnosed through either clinic or imaging). In case of unknown event status at last follow-up date, data were considered missing. Patients died of an intercurrent disease or an unspecified reason were not considered in DSS analyses.

### Ultrasound

All transvaginal ultrasound examinations were performed using a Voluson™ E6 (GE Healthcare, Chicago, Illinois, United States) equipped with a multifrequency endovaginal probe (4.0 to 9.0 MHz). The probe was introduced into the posterior vaginal fornix, and the uterus was studied in sagittal and transversal section. The tumor was evaluated by two-dimensional gray-scale ultrasound. The three maximum orthogonal diameters of the tumor were recorded and the maximum diameter was used for analysis.

### Data collection

Collected data included patient age, menopausal status, body mass index (BMI), history of abnormal uterine bleeding (AUB), hypertension, diabetes, previous use of tamoxifen, FIGO stage, grade, histotype, LVSI, myometrial invasion, lymph nodes involvement and adjuvant treatment.

### Statistical analysis

Numerical and categorical variables were summarized as median [range] and as frequencies and percentages, respectively.

Differences in the distribution of classic prognostic factors (i.e. age >70 years, myometrial invasion, cervical stromal invasion, LVSI, and lymph node involvement) between groups of patients based on tumor diameter were evaluated using the chi-squared test or Fisher’s exact test, where appropriate. We used the Kaplan–Meier estimator to display OS, DSS and PFS in the two groups; the equality of survivor functions was assessed using the log-rank test. The same analysis was repeated according to age (≤70, >70 years), BMI (<25, 25–29.9,≥30 kg/m²), FIGO stage, FIGO grade (1-2; 3), LVSI status (LVS no, LVS yes), myometrial invasion (<50%, >50%), lymph nodal involvement (no, yes), histotype (endometrioid, non-endometrioid), adjuvant treatment (no, yes).

If an association was found between tumor size and survival outcomes, a Cox proportional hazards model including the propensity score of belonging to one of the two groups given the set of baseline potential confounders was planned to analyze the adjusted association between tumor size and survival. Effect sizes were expressed as hazard ratios (HRs) and 95% confidence intervals (CIs).

All analyses were carried out using Stata software, version 15 (StataCorp, 2017, Stata Statistical Software: Release 15, College Station, Texas, USA: StataCorp LP). The significance level was set at 5%.

### Ethical statement

The study received approval by the Institutional Review Board of the IRCCS Azienda Ospedaliero-Universitaria di Bologna, S. Orsola Hospital, University of Bologna, Italy (No.: 429/2021/Oss/AOUBo) and was carried out according to the principles of the Declaration of Helsinki. All patients signed a written informed consent, and all data were anonymized.

## Results

### Study population

A total of 108 patients meeting selection criteria were included in the study. Characteristics of the study population are summarized in **Table 1**, while the distribution of histological prognostic factors both overall and by tumor size, is shown in **Table 2**.

**Table 1:** Characteristics of the study population (n = 108).

Characteristic	
Age, years	68 [35-90]
Body mass index, kg/m^2^	27.0 [19.5-49.0]
Presence of Abnormal Uterine Bleeding	99 (91.7)
Diabetes	17 (15.7)
Hypertension	61 (56.5)
FIGO stage	
IA	61 (56.5)
IB	16 (14.8)
II	2 (1.9)
IIIA	0 (0.0)
IIIB	0 (0.0)
IIIC1	16 (14.8)
IIIC2	13 (12.0)
Grade	
Grade 1	21 (19.5)
Grade 2	74 (68.5)
Grade 3	13 (12.0)
Histotype	
Endometrioid	102 (94.4)
Non-endometrioid	6 (5.6)
Mean tumor size	3.3 cm
Type of surgery (Total Hysterectomy with BSO)	
Laparoscopic	78 (72.2)
Abdominal	30 (27.8)
Evaluation of LN status during surgery	
Sentinel LN	48 (44.4)
Systematic Lymphadenectomy	60 (55.6)
Pelvic	47 (78.3)
Pelvic and Lombo-Aortic	13 (21.7)

Data are presented as median [range] for continuous variables and as n (%) for categorical variables. FIGO, International Federation of Gynecology and Obstetrics; BSO, bilateral salpingo-oophorectomy; LN, lymph node.

**Table 2:** Distribution of histological prognostic factors in the study population, overall and by tumor size.

Prognostic factor	All (n = 108)	Tumor size								
		<2 cm	≥2 cm	p	<3 cm	≥3 cm	p	<4 cm	≥4 cm	p
		(n = 26)	(n = 82)		(n = 43)	(n = 65)		(n = 83)	(n = 25)	
Age >70 y	45 (41.7%)	10 (38.5%)	35 (42.7%)	0.704	17 (39.5%)	28 (43.1%)	0.715	34 (41.0%)	11 (44.0%)	0.787
Deep myometrial invasion	100 (92.6%)	22 (84.6%)	78 (95.1%)	0.093	38 (88.4%)	62 (95.4%)	0.261	76 (91.6%)	24 (96.0%)	0.678
Cervical stromal invasion	11 (10.2%)	0 (0.0%)	11 (13.4%)	0.063	1 (2.3%)	10 (15.4%)	0.047*	6 (7.2%)	5 (20.0%)	0.123
Lymph-vascular space invasion	57 (52.8%)	9 (34.6%)	48 (58.5%)	0.033*	15 (34.9%)	42 (64.6%)	0.002*	39 (47.0%)	18 (72.0%)	0.028*
Lymph node involvement	29 (26.9%)	5 (19.2%)	24 (29.3%)	0.314	8 (18.6%)	21 (32.3%)	0.116	19 (22.9%)	10 (40.0%)	0.091

*P value ≤0.05.

All patients were diagnosed with EC by hysteroscopic endometrial biopsy. Regarding surgical treatment, 78 patients (72.2%) underwent laparoscopic surgery, while 30 patients (27.8%) underwent laparotomic surgery. Systematic lymphadenectomy was performed in 60 patients (55.6%), respectively 47 patients (78.3%) underwent pelvic lymphadenectomy and 13 patients (21.7%) pelvic and lombo-aortic lymphadenectomy. Lymph node metastasis were reported in 29 cases (26.9%). Sentinel lymph node biopsy was performed in 48 (44.4%) cases and metastasis were found in 4 (3.7%) patients (**Table 1**).

According to the tumor size, 26 patients (24.1%) were included in the group with <2 cm tumor, 82 (75.9%) in ≥2 cm group, 43 (39.8%) in <3 cm group, 65 (60.2%) in ≥3 cm group, 83 (76.8%) in <4 cm group and 25 (23.2%) in ≥4 cm group. Among classic prognostic factors, LVSI was significantly more frequent in ≥2, ≥3 and ≥4 cm groups compared to <2, <3 and <4 cm groups, respectively (**Table 2**).

### Survival analyses

The cumulative incidence was 18.5% for death of any cause, 6.5% for death due to EC and 14.8% for disease recurrence. The incidence density rates were 5.1×100, 1.8×100 and 4.3×100 person-years, respectively. Eighty-eight patients were alive at the time of this analysis, with a median follow-up of 50 months (47 months if extended to the whole sample).

OS, DSS and PFS did not significantly differ between the groups based on the different tumor diameter cut-offs (**Figures 1**–**3**). No significant differences in OS, DSS and PFS were found among the groups sub-stratified by age, BMI, FIGO stage, FIGO grade, LVSI status, myometrial invasion, lymph nodal involvement, histotype, and adjuvant treatment (**Supplementary Figures 1**–**9**).

**Figure 1:**
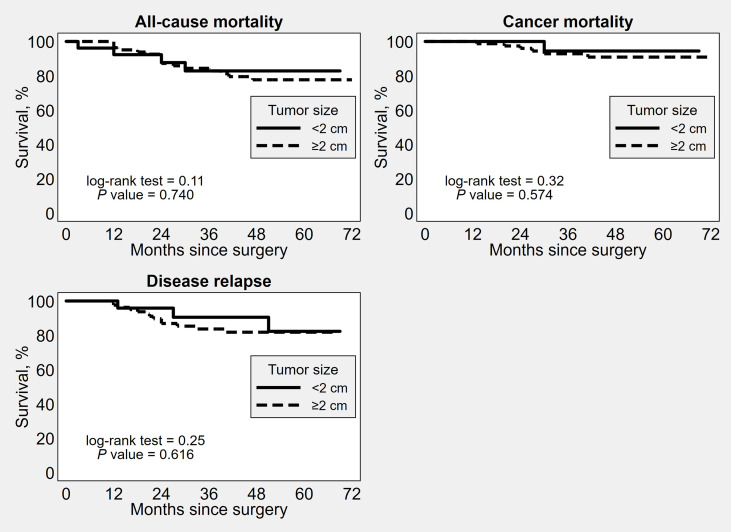
Kaplan–Meier survival estimates of time to all-cause mortality, cancer mortality and disease recurrence after surgery, by 2 cm tumor diameter cut-off.

**Figure 2:**
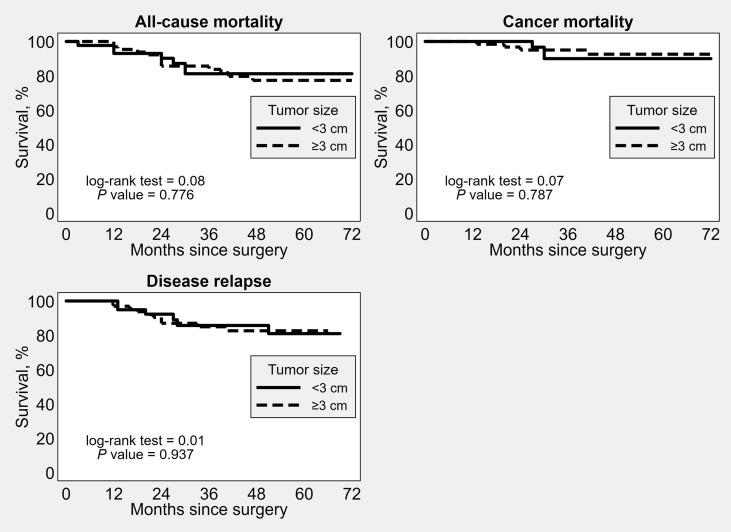
Kaplan–Meier survival estimates of time to all-cause mortality, cancer mortality and disease recurrence after surgery, by 3 cm tumor diameter cut-off.

**Figure 3:**
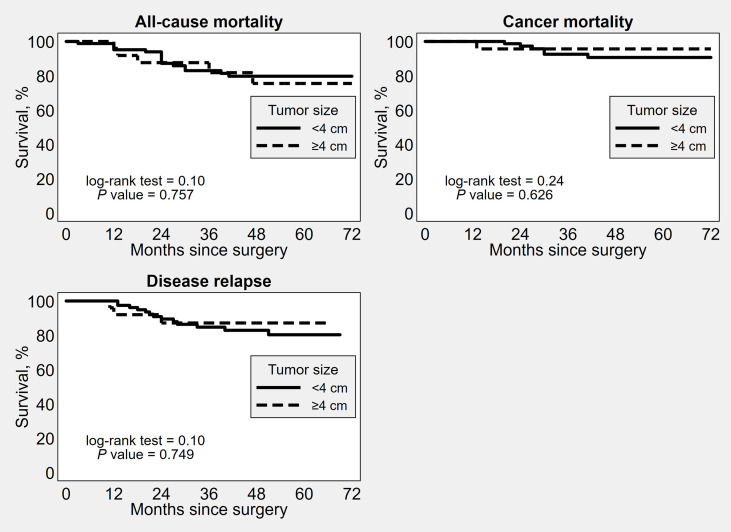
Kaplan–Meier survival estimates of time to all-cause mortality, cancer mortality and disease recurrence after surgery, by 4 cm tumor diameter cut-off.

## Discussion

### Main findings and interpretation

This study shows that preoperative ultrasound tumor size does not appear as a prognostic factor for death of any cause, death due to EC and recurrence in EC patients. Moreover, no significant differences in survival analyses were found among the groups sub-stratified by other prognostic factors.

In accordance with the principles of the precision medicine (4–6) and even more after the increase in number of deaths per year reported in the last decades in EC patients (20), an increasingly tailored and accurate risk assessment appears crucial. New prognostic factors to be investigated may be useful to refine the current risk stratification system. Beyond the TCGA molecular advances, the tumor size remains the only prognostic factor to be further assessed among the histological ones.

Tumor size has shown prognostic significance in several malignancies, such as lung, breast and ovarian granulosa cell tumors (7, 8, 21). However, its value is unclear in EC patients. In particular, while some authors found a significant association between tumor size and some histological prognostic factors, its impact on survival outcomes was uncertain (9, 10, 13, 22). Berretta et al. found a significant difference in size between FIGO stage IA (mean diameter 2,9 cm) and stage IB (mean diameter 4,4 cm) ECs, showing an increased risk of deep myometrial invasion and LVSI in tumor greater than 3 cm (10). On the other hand, Laufer et al. showed that even tumors greater than 2 cm were associated with an increased risk of deep myometrial invasion, low FIGO grade and LVSI (21). Furthermore, tumor size has also been associated with lymph node involvement. Boyraz et al. reported that a tumor size greater than 2 cm might be considered an independent predictor of lymph node metastasis in patients with low-risk EC (9). Mariani et al. reported no lymph node metastases among patients with primary tumor diameter ≤2 cm (12). A similar conclusion was reached by Vargas et al. assessing data from the National Cancer Institute’s Surveillance, Epidemiology, and End Results Program (SEER) registry. In particular, they found that lymph node involvement rate increased from 1.3% in grade 1 and 3.8% in grade 2 tumors ≤2 cm to 12.7% in grade 1 and 23% in grade 2 tumors ≥ 5 cm, independently of myometrial invasion. The increased risk of node metastasis was also confirmed at multivariate analysis (23). In another study Cox-Bauer et al. reported that a cut-off of 5 cm was significantly more predictive of nodal involvement than a tumor diameter of 2 cm (11).

Concerning the impact of tumor size on survival outcomes, conflicting results have been reported in the Literature. Some Authors reported tumor size as an independent prognostic factor for recurrence alone (19, 24) or for recurrence and death due to EC (25); other Authors did not confirm an independent association between tumor size and recurrence (14, 15, 26). In particular, Chattopadhyay S. et al. found that a tumor size cut-off of 3.75 cm could be considered a significant independent prognostic factor of death due to EC and recurrence in FIGO Stage I EC patients who did not undergo lymphadenectomy (25). Senol T. et al. showed that the same cut-off was a predictor for recurrence, but not for death of any cause (p >0.05) (19). The association between tumor size and recurrence was found even with a smaller cut-off (i.e. 2.5 cm) in low-risk EC patients according to the European Society of Medical Oncology-European Society of Gynecological Oncology-European Society for Radiotherapy and Oncology classification (24). On the contrary, other studies showed that, although there was an increased risk of nodal metastasis in patients with tumors >2 cm, tumor size did not appear as an independent predictor of recurrence (15, 26). In another study, the association with recurrence was not confirmed neither considering a cut-off of 3.5 cm (14).

Beyond the conflicting findings, previous studies have focused on tumor size at histological examination. In our study, conversely, we focused on the tumor diameter at ultrasound. In fact, this could improve the preoperative risk stratification of EC patients. Although preoperative ultrasound tumor size was associated with LVSI, we found that it was not a prognostic factor for death of any cause, death due to EC and recurrence in EC patients. These findings were confirmed even adopting different tumor diameter cut-offs (i.e. 2, 3 and 4 cm). Our results suggest that ultrasound tumor size does not appear as an additional prognostic factor to further refine the preoperative risk stratification of EC patients. However, further studies are needed to confirm these findings.

### Strengths and limitations

To our knowledge, our study may be the first study to assess the prognostic value of tumor size in EC patients at preoperative ultrasound. In fact, the impact of tumor size on cancer outcomes has been mainly assessed at postoperative histological examination so far, with only few studies assessing its prognostic role preoperatively on magnetic resonance imaging (27, 28). Having an additional preoperative prognostic factor might help plan surgical staging and further refine risk stratification and management of EC patients.

A major limitation of our study underlies in the retrospective design which affects data availability. However, missing data from medical records and clinical electronic databases did not affect our main analyses. Moreover, the inclusion of patients from a single center minimized the biases arising from different patient management and data collection. Another important limitation of our study may be that we didn’t assess postoperative pathological tumor size in addition to preoperative ultrasound tumor size. Anyway, transvaginal ultrasound has been established as an effective tool to evaluate endometrial pathology (29–31). Lastly, as a further limitation, we were unable to assess tumor size as a prognostic factor in each TCGA molecular group. In fact, like other histological factors (32–36), it might have a prognostic role only in selected TCGA groups.

## Conclusions

Preoperative ultrasound tumor size does not appear as a prognostic factor for death of any cause, death due to EC and recurrence in EC women. Its assessment does not seem to be useful to further refine the preoperative risk stratification of patients. Further studies are needed to confirm these findings.

## Data availability statement

The raw data supporting the conclusions of this article will be made available by the authors, without undue reservation.

## Ethics statement

The studies involving human participants were reviewed and approved by Institutional Review Board of the IRCCS Azienda Ospedaliero-Universitaria di Bologna, S. Orsola Hospital, University of Bologna, Italy (No.: 429/2021/Oss/AOUBo). The patients/participants provided their written informed consent to participate in this study.

## Author contributions

Conceptualization: MA and PC; methodology: AAr, AR, DN, MF, AM, AT, and RP; software: AAl and AAr; validation: RP, AT, PC, and MG; formal analysis: DR, FF, and PS; investigation: AAl, DN, MF, CC, AT, and FF; resources: AAl, AR, MA, and MG; data curation: MA, AR, DR, CC, and AAl; writing-original draft preparation: MA, AR, DN, MF, and CC, writing-review and editing: MA, AAl, AR; visualization: MA, AR, DN, MF, AAr, AT, and AM; supervision: AM, RP, AM, PC, and RS; project administration: MA and PC. All authors contributed to the article and approved the submitted version.

## Funding

The work reported in this publication was funded by the Italian Ministry of Health, RC-2022-n.2773472.

## Conflict of interest

The authors declare that the research was conducted in the absence of any commercial or financial relationships that could be construed as a potential conflict of interest.

## Publisher’s note

All claims expressed in this article are solely those of the authors and do not necessarily represent those of their affiliated organizations, or those of the publisher, the editors and the reviewers. Any product that may be evaluated in this article, or claim that may be made by its manufacturer, is not guaranteed or endorsed by the publisher.

## Supplementary material

Supplementary Figure 1 Kaplan–Meier survival estimates of time to all-cause mortality, for prognostic group (stage, grade, age, BMI, stage, grade, LVSI, myometrial invasion, lymph node involvement, histotype, adjuvant treatment) and by 2 cm tumor diameter cut-off.

Supplementary Figure 2 Kaplan–Meier survival estimates of time to death from cancer, for prognostic group (stage, grade, age, BMI, stage, grade, LVSI, myometrial invasion, lymph node involvement, histotype, adjuvant treatment) and by 2 cm tumor diameter cut-off.

Supplementary Figure 3 Kaplan–Meier survival estimates of time to disease relapse, for prognostic group (stage, grade, age, BMI, stage, grade, LVSI, myometrial invasion, lymph node involvement, histotype, adjuvant treatment) and by 2 cm tumor diameter cut-off.

Supplementary Figure 4 Kaplan–Meier survival estimates of time to all-cause mortality, for prognostic group (stage, grade, age, BMI, stage, grade, LVSI, myometrial invasion, lymph node involvement, histotype, adjuvant treatment) and by 3 cm tumor diameter cut-off.

Supplementary Figure 5 Kaplan–Meier survival estimates of time to death from cancer, for prognostic group (stage, grade, age, BMI, stage, grade, LVSI, myometrial invasion, lymph node involvement, histotype, adjuvant treatment) and by 3 cm tumor diameter cut-off.

Supplementary Figure 6 Kaplan–Meier survival estimates of time to disease relapse, for prognostic group (stage, grade, age, BMI, stage, grade, LVSI, myometrial invasion, lymph node involvement, histotype, adjuvant treatment) and by 3 cm tumor diameter cut-off.

Supplementary Figure 7 Kaplan–Meier survival estimates of time to all-cause mortality, for prognostic group (stage, grade, age, BMI, stage, grade, LVSI, myometrial invasion, lymph node involvement, histotype, adjuvant treatment) and by 4 cm tumor diameter cut-off.

Supplementary Figure 8 Kaplan–Meier survival estimates of time to death from cancer, for prognostic group (stage, grade, age, BMI, stage, grade, LVSI, myometrial invasion, lymph node involvement, histotype, adjuvant treatment) and by 4 cm tumor diameter cut-off.

Supplementary Figure 9 Kaplan–Meier survival estimates of time to disease relapse, for prognostic group (stage, grade, age, BMI, stage, grade, LVSI, myometrial invasion, lymph node involvement, histotype, adjuvant treatment) and by 4 cm tumor diameter cut-off.
