# Supplementary figures and images for: Is preoperative ultrasound tumor size a prognostic factor in endometrial carcinoma patients?
Source: Front Oncol. 2022 Sep 23;12:993629. doi: 10.3389/fonc.2022.993629 (PMC9538669; doi:10.3389/fonc.2022.993629)

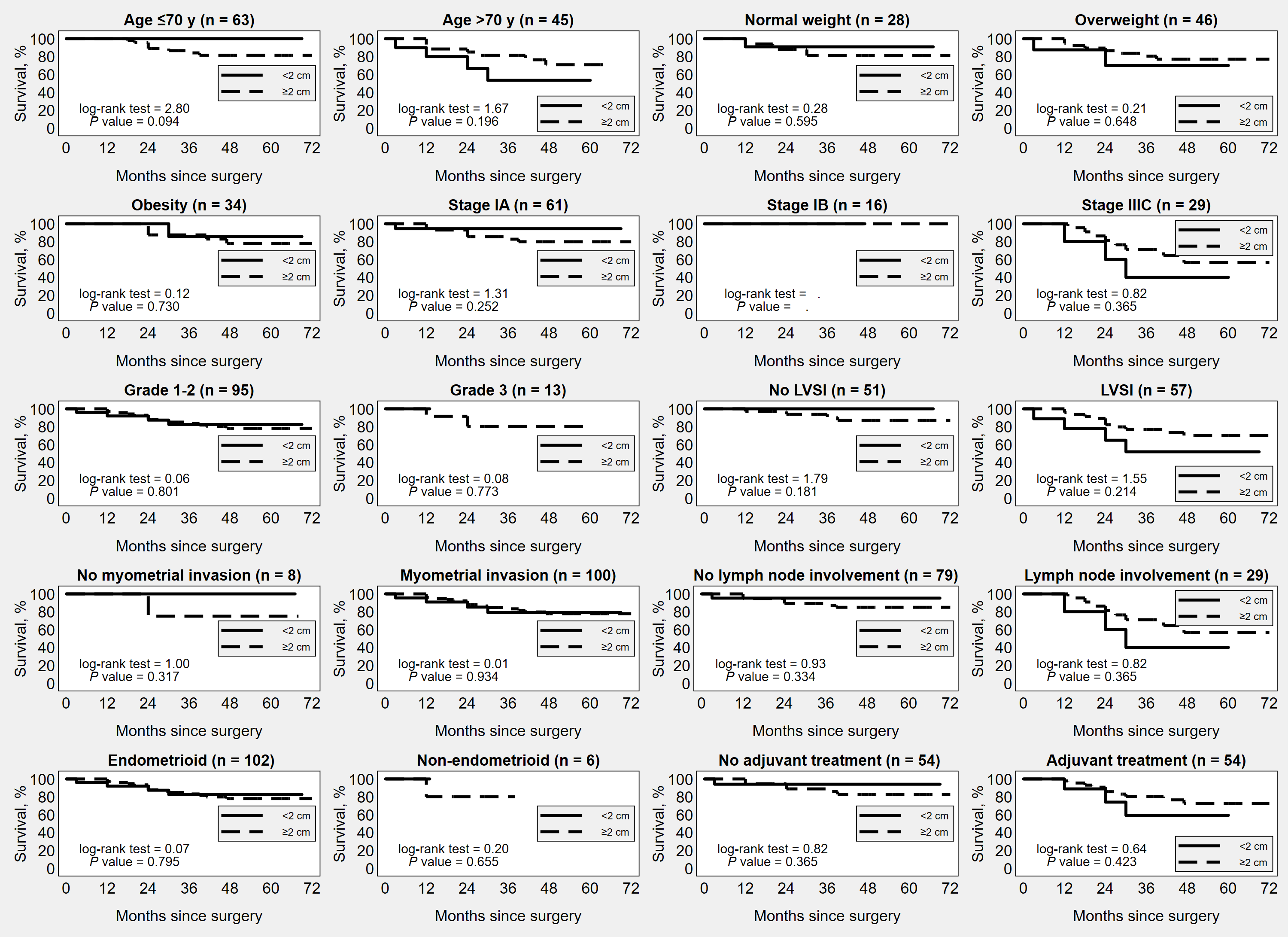

Supplement: Supplementary Figure 1 — Kaplan–Meier survival estimates of time to all-cause mortality, for prognostic group (stage, grade, age, BMI, stage, grade, LVSI, myometrial invasion, lymph node involvement, histotype, adjuvant treatment) and by 2 cm tumor diameter cut-off. [file Image_1.tif]

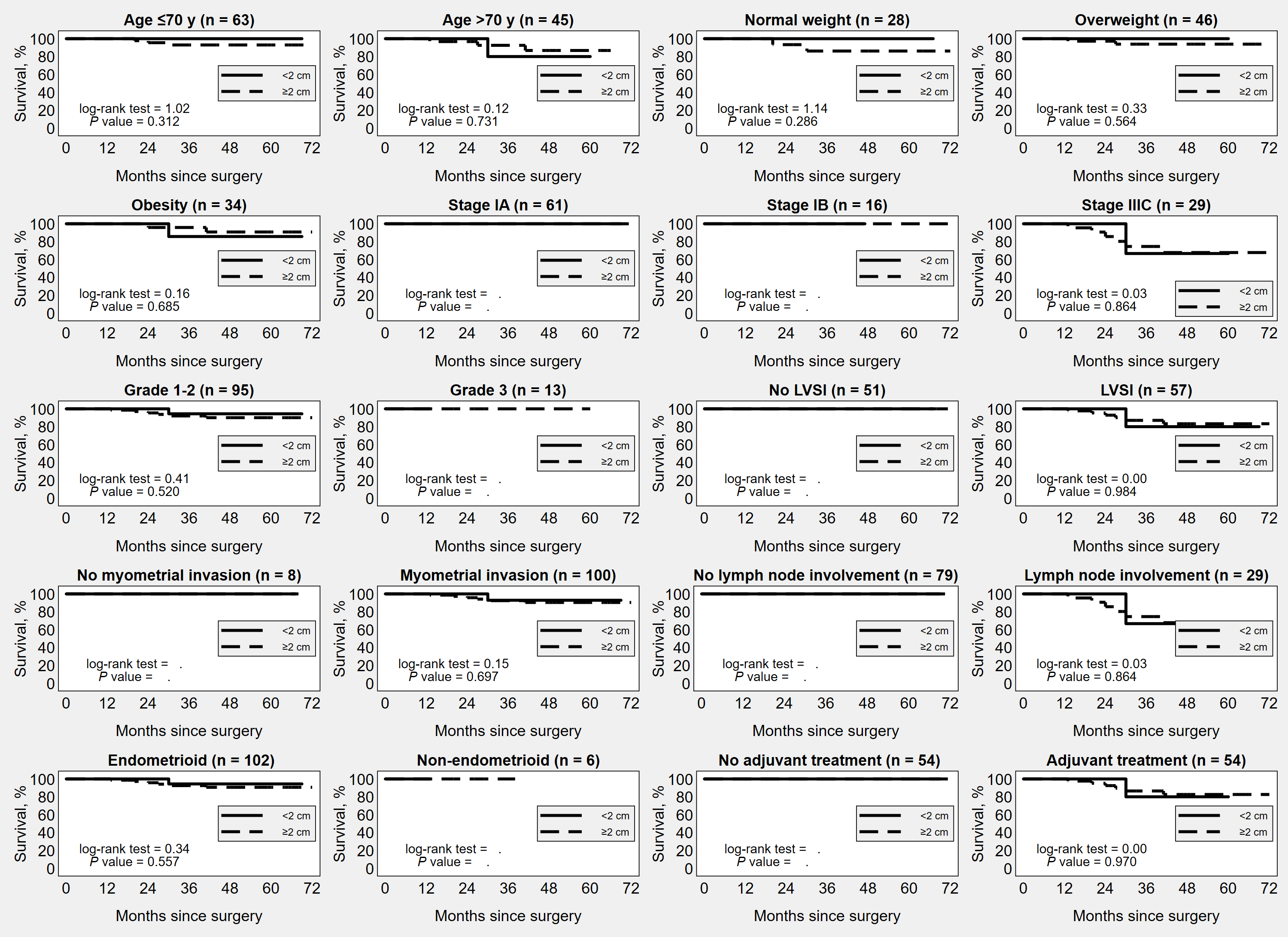

Supplement: Supplementary Figure 2 — Kaplan–Meier survival estimates of time to death from cancer, for prognostic group (stage, grade, age, BMI, stage, grade, LVSI, myometrial invasion, lymph node involvement, histotype, adjuvant treatment) and by 2 cm tumor diameter cut-off. [file Image_2.tif]

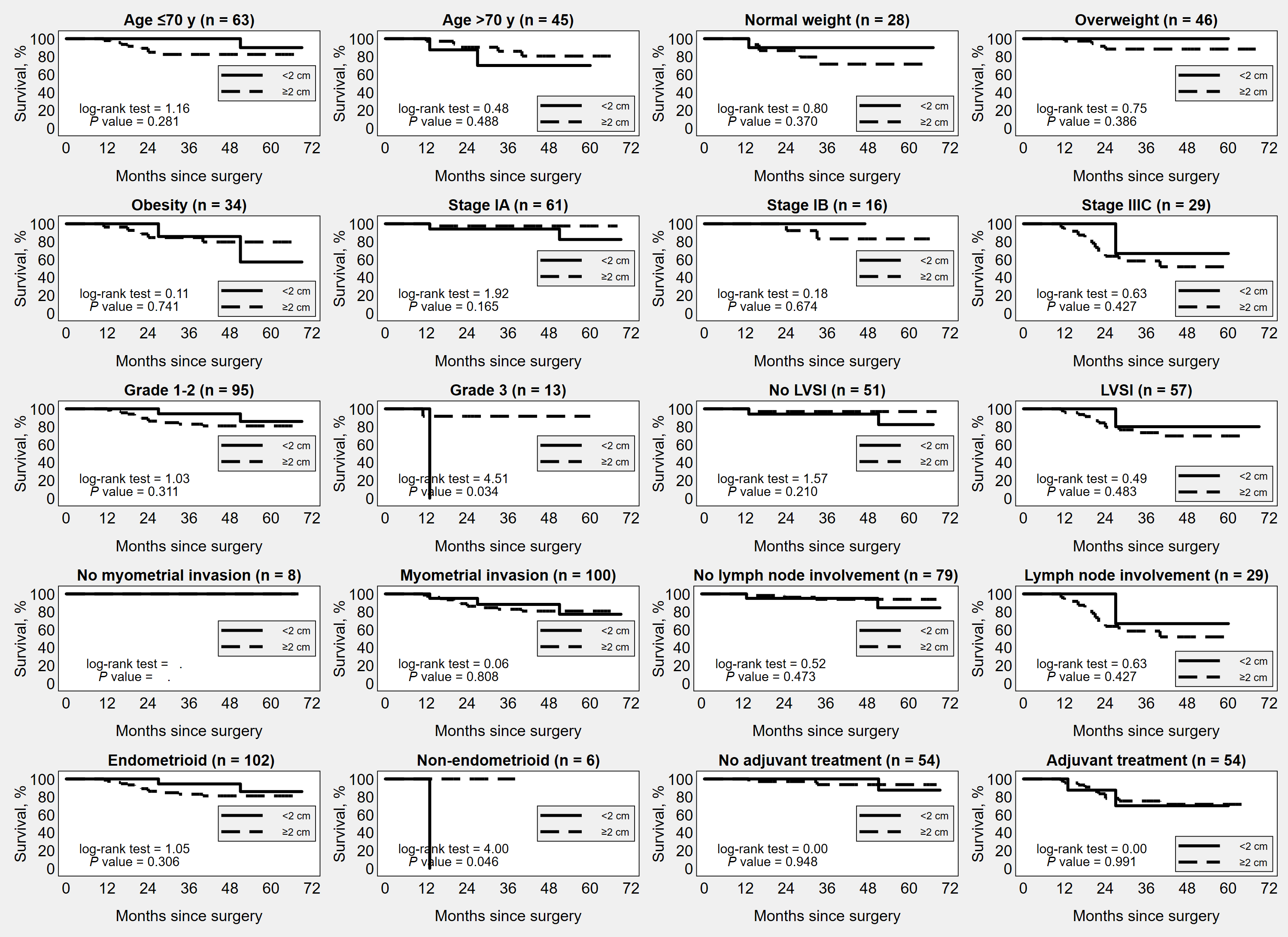

Supplement: Supplementary Figure 3 — Kaplan–Meier survival estimates of time to disease relapse, for prognostic group (stage, grade, age, BMI, stage, grade, LVSI, myometrial invasion, lymph node involvement, histotype, adjuvant treatment) and by 2 cm tumor diameter cut-off. [file Image_3.tif]

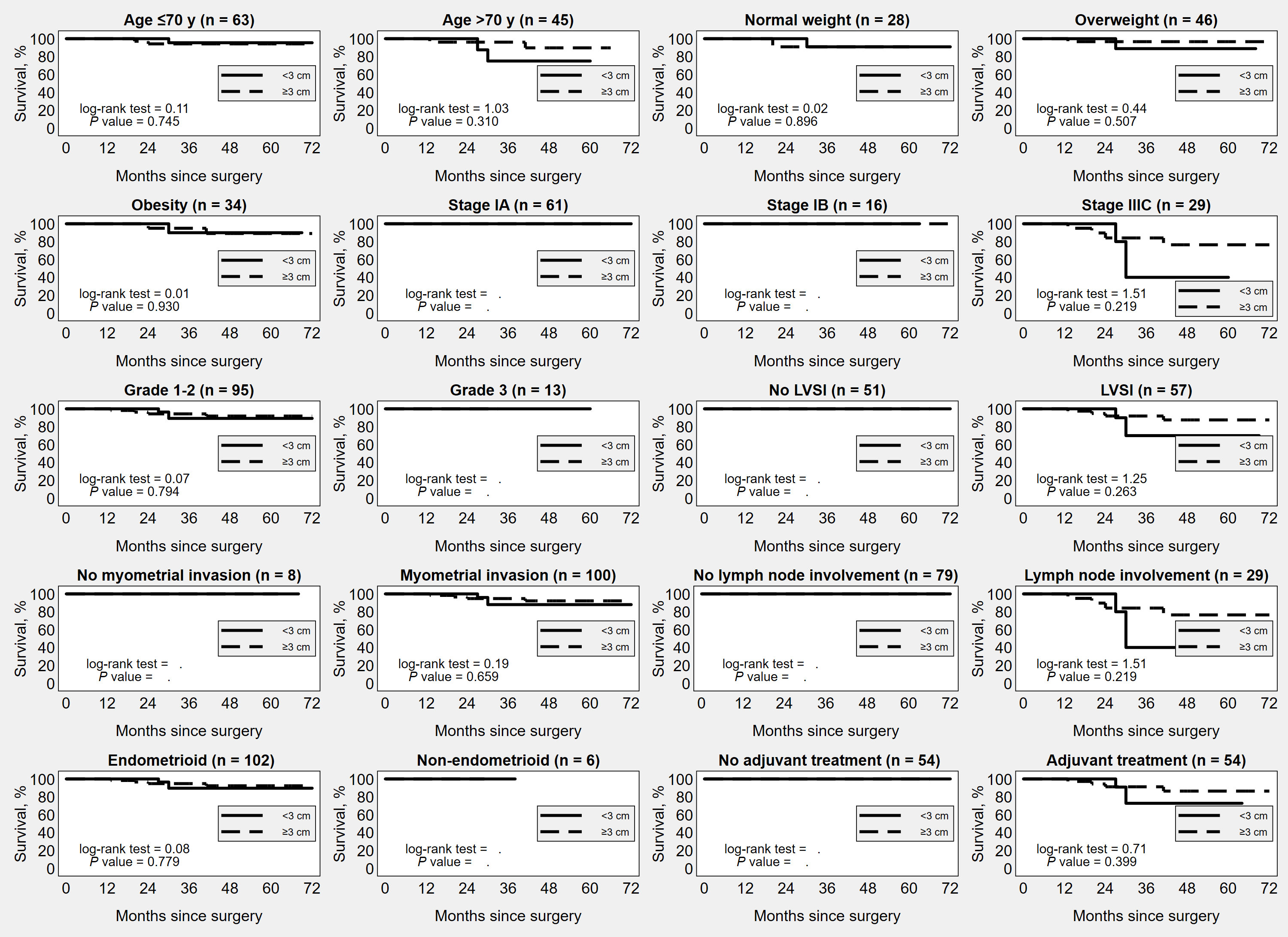

Supplement: Supplementary Figure 4 — Kaplan–Meier survival estimates of time to all-cause mortality, for prognostic group (stage, grade, age, BMI, stage, grade, LVSI, myometrial invasion, lymph node involvement, histotype, adjuvant treatment) and by 3 cm tumor diameter cut-off. [file Image_4.tif]

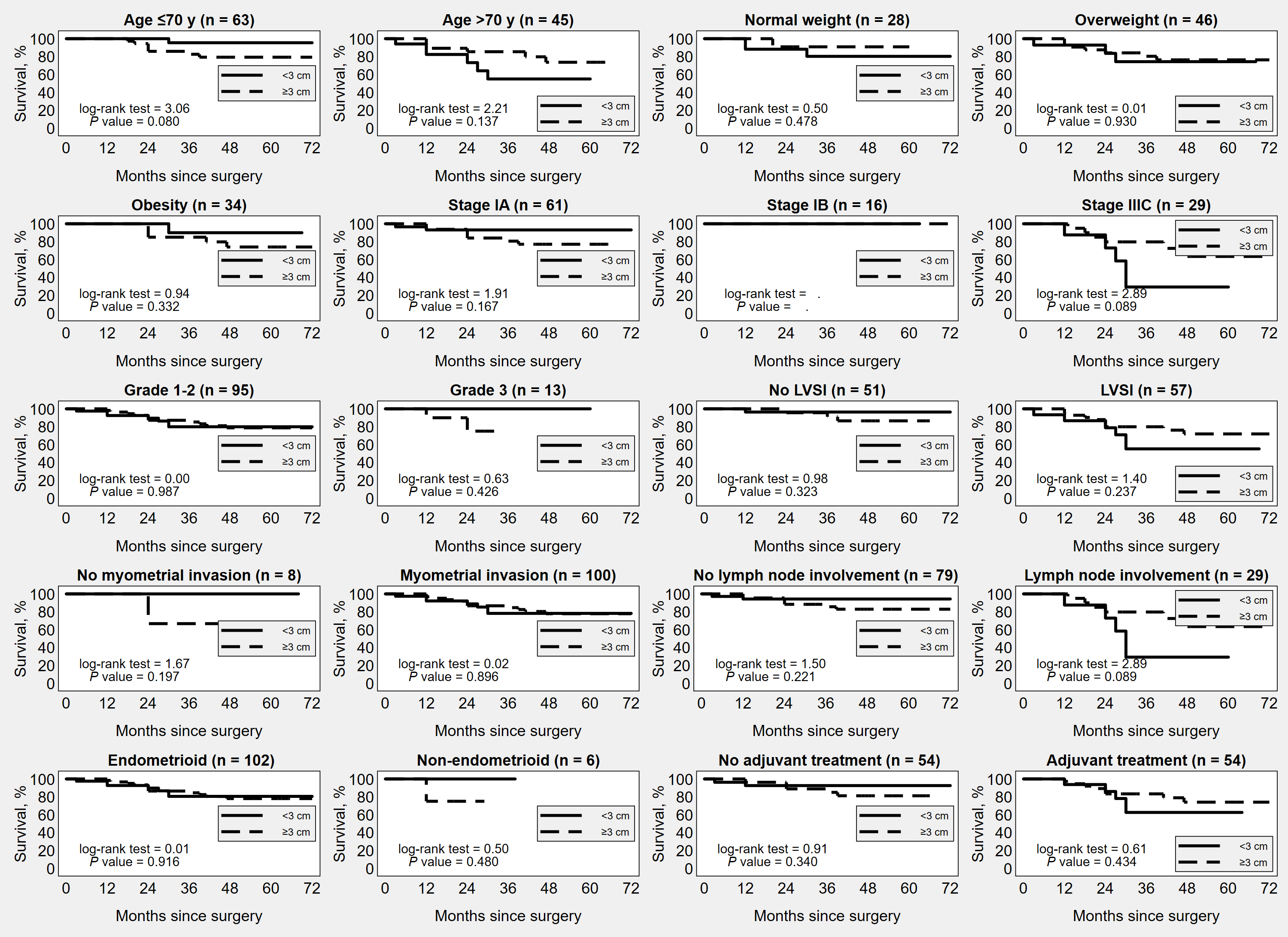

Supplement: Supplementary Figure 5 — Kaplan–Meier survival estimates of time to death from cancer, for prognostic group (stage, grade, age, BMI, stage, grade, LVSI, myometrial invasion, lymph node involvement, histotype, adjuvant treatment) and by 3 cm tumor diameter cut-off. [file Image_5.tif]

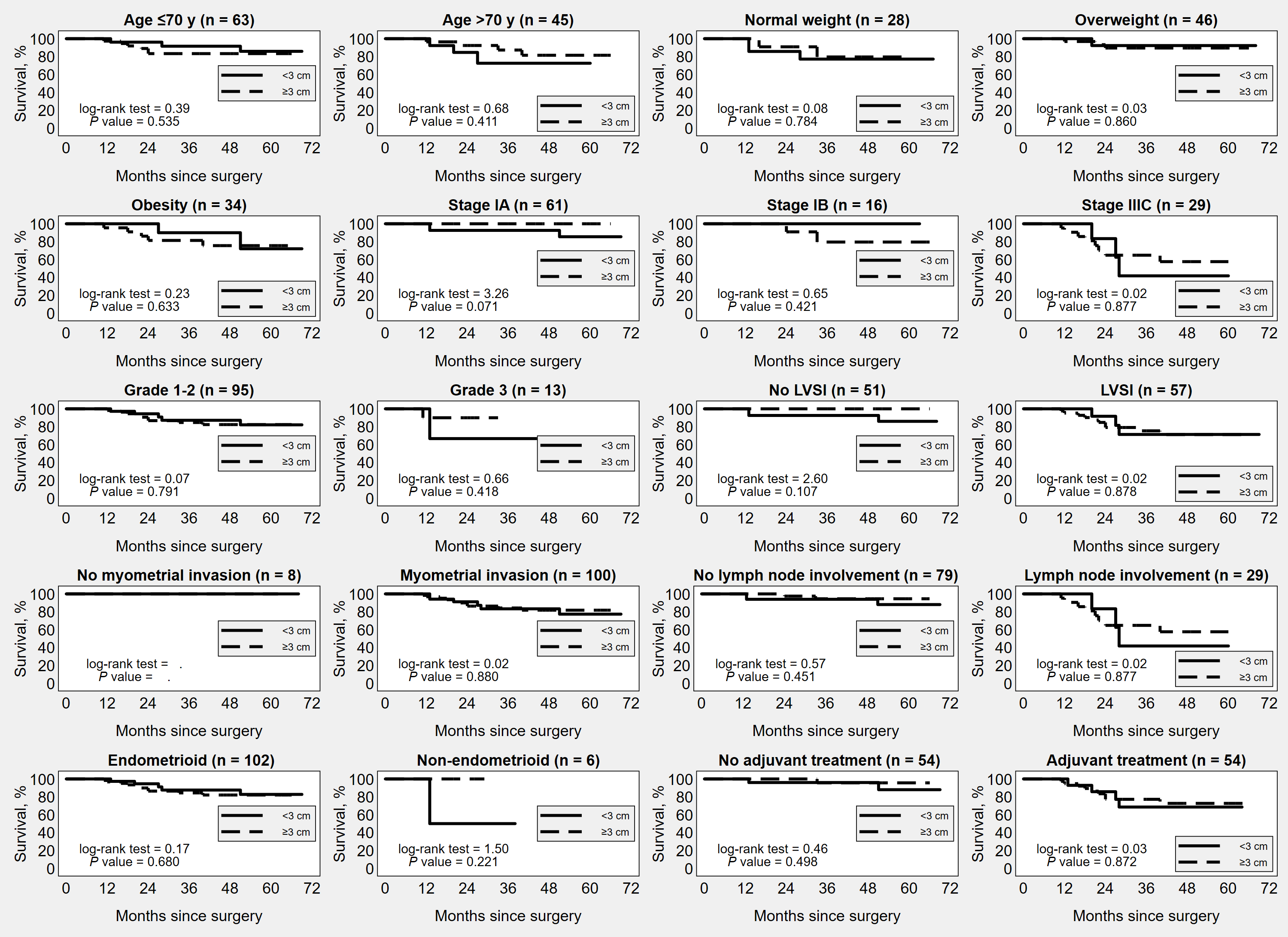

Supplement: Supplementary Figure 6 — Kaplan–Meier survival estimates of time to disease relapse, for prognostic group (stage, grade, age, BMI, stage, grade, LVSI, myometrial invasion, lymph node involvement, histotype, adjuvant treatment) and by 3 cm tumor diameter cut-off. [file Image_6.tif]

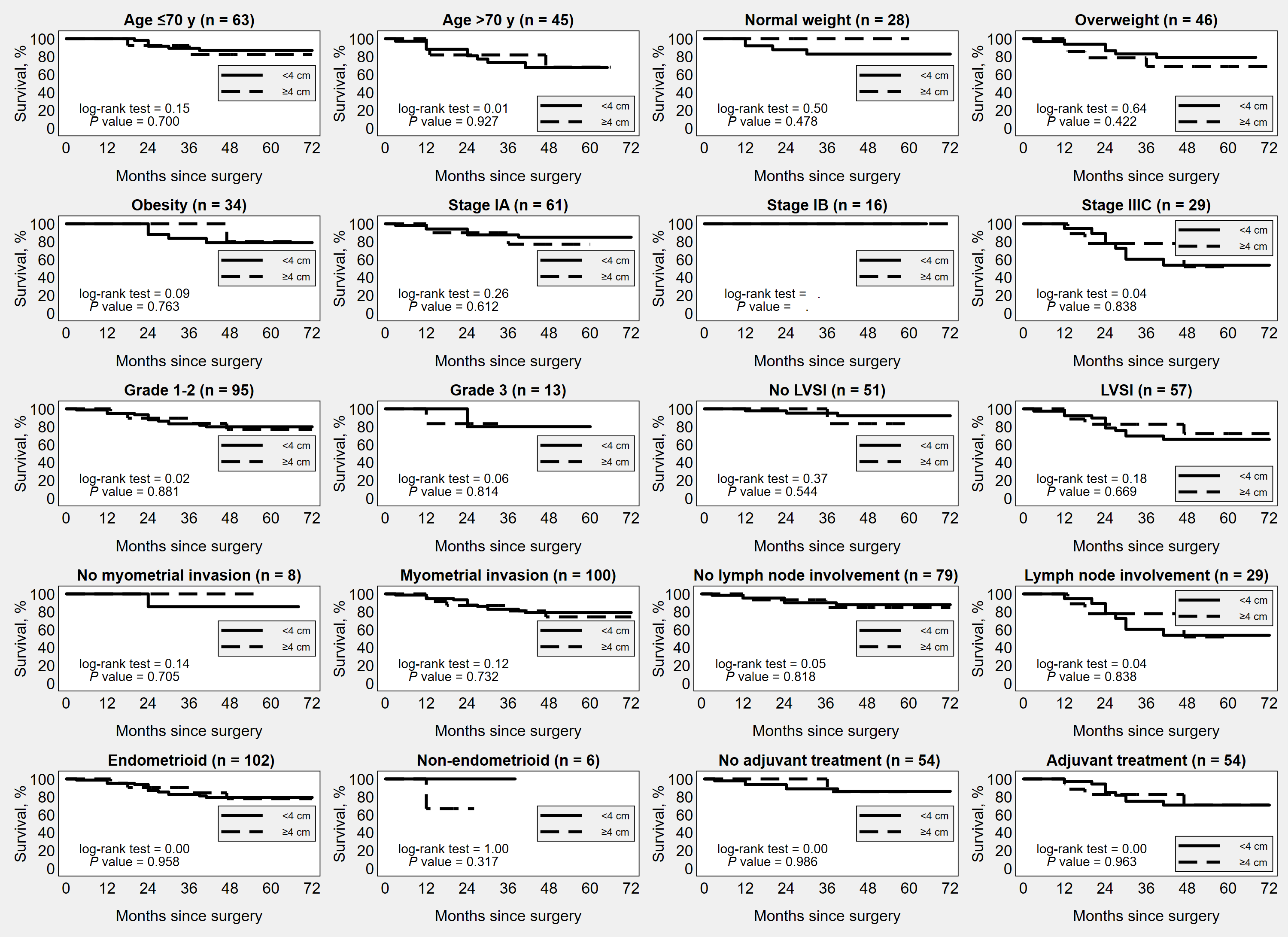

Supplement: Supplementary Figure 7 — Kaplan–Meier survival estimates of time to all-cause mortality, for prognostic group (stage, grade, age, BMI, stage, grade, LVSI, myometrial invasion, lymph node involvement, histotype, adjuvant treatment) and by 4 cm tumor diameter cut-off. [file Image_7.tif]

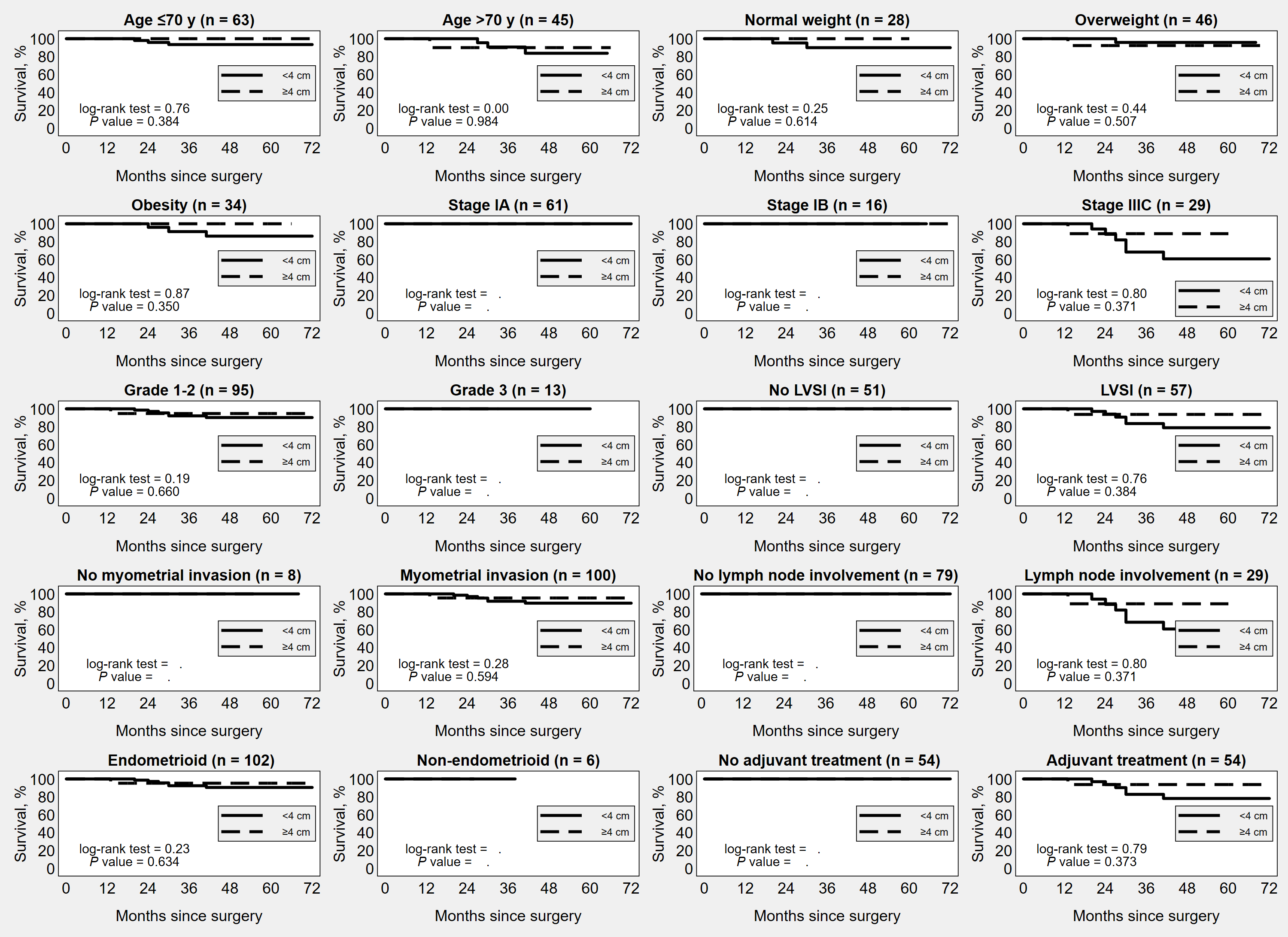

Supplement: Supplementary Figure 8 — Kaplan–Meier survival estimates of time to death from cancer, for prognostic group (stage, grade, age, BMI, stage, grade, LVSI, myometrial invasion, lymph node involvement, histotype, adjuvant treatment) and by 4 cm tumor diameter cut-off. [file Image_8.tif]

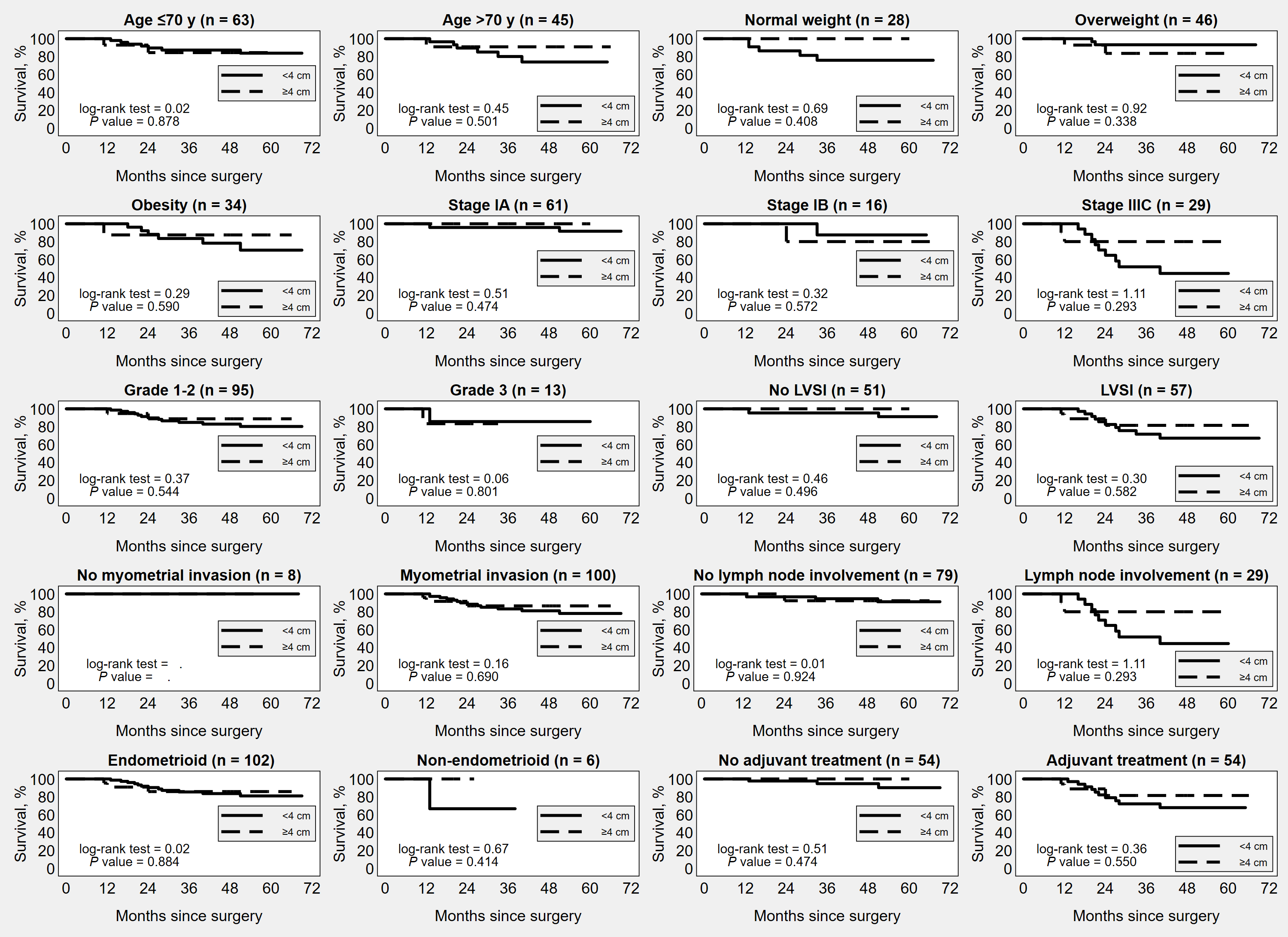

Supplement: Supplementary Figure 9 — Kaplan–Meier survival estimates of time to disease relapse, for prognostic group (stage, grade, age, BMI, stage, grade, LVSI, myometrial invasion, lymph node involvement, histotype, adjuvant treatment) and by 4 cm tumor diameter cut-off. [file Image_9.tif]
